# Supplementary material for: X-ray linear dichroic tomography of crystallographic and topological defects
Source: Nature. 2024 Dec 11;636(8042):354–60. doi: 10.1038/s41586-024-08233-y (PMC11634779; doi:10.1038/s41586-024-08233-y)
Supplement: Supplementary file 1 — This file contains Supplementary Notes 1–3, Supplementary Figs. 1–14, Supplementary Table 1 and Supplementary References [file 41586_2024_8233_MOESM1_ESM.docx]

**Supplementary Information**

This Supplementary Information Includes:

- Supplementary Note 1-3
- Supplementary Figure 1-14
- Supplementary Table 1
- Supplementary References

References:

91. Fu, J. & Urquhart, S. G. Linear Dichroism in the X-ray Absorption Spectra of Linear *n* -Alkanes. *J. Phys. Chem. A* **109**, 11724–11732 (2005).

92. Wu, Q.-H., Thissen, A., Jaegermann, W. & Liu, M. Photoelectron spectroscopy study of oxygen vacancy on vanadium oxides surface. *Applied Surface Science* **236**, 473–478 (2004).

93. Zhou, D. *et al.* Assignment of Polarization-Dependent Peaks in Carbon K-Edge Spectra from Biogenic and Geologic Aragonite. *J. Phys. Chem. B* **112**, 13128–13135 (2008).

94. Fevola, G. *et al.* Resonant x-ray ptychographic nanotomography of kesterite solar cells. *Phys. Rev. Research* **2**, 013378 (2020).

95. Maddali, S. *et al.* Concurrent multi-peak Bragg coherent x-ray diffraction imaging of 3D nanocrystal lattice displacement via global optimization. *npj Comput Mater* **9**, 77 (2023).

96. Ishiguro, N. & Takahashi, Y. Method for restoration of X-ray absorption fine structure in sparse spectroscopic ptychography. *J Appl Crystallogr* **55**, 929–943 (2022).

97. McCusker, L. B., Von Dreele, R. B., Cox, D. E., Louër, D. & Scardi, P. Rietveld refinement guidelines. *J Appl Crystallogr* **32**, 36–50 (1999).

98. Popa, N. C. The (hkl) Dependence of Diffraction-Line Broadening Caused by Strain and Size for all Laue Groups in Rietveld Refinement. *J. Appl. Crystallogr.* **31**, 176–180 (1998).

**Supplementary Note 1: X-ray linear dichroism as reflected in the pre-edge peak intensity of V_2_O_5_:** X-ray linear dichroism (XLD) is a measure of the local changes of a material's refractive index as a function of incident beam polarisation and energy.^29^ Specifically, XLD, refers to the anisotropic absorption of linearly polarised electromagnetic radiation due to the orientation of a sample feature relative to the electric field vector of the radiation ^16,91^. XLD contrast is present in the X-ray absorption near-edge structure (XANES) region for photon energies in the pre-edge peak, providing sensitivity to the coordination geometry of the probed chemical element. The polarisation and sample orientation-dependent absorption cross-section in XLD measurements allows for the determination of the spatial orientation of the probed chemical bonds. Multiple measurements at the pre-edge peak energy, in this case 5.469 eV as a function of the linear polarisation state of the illumination, allows the determination of the orientation of the probed chemical bonds^16,91^ The intensity, *I*, of near-edge features, including the pre-peak intensity, depends as explained in Fu et. al. (2005)^91^ on the angle, $\varphi$, between the electric field vector (in other words, the linear polarisation vector of the illumination) and the angle-integrated absorption cross-section, A (Equation S1).

| $I\approx A{cos}^{2} \varphi$ | (S1) |
| --- | --- |

A pre-requisite for any linear dichroism is the presence of an anisotropic “bonding environment”. The V_2_O_5_ examined here satisfies this pre-requisite due to the distorted [VO_5_] pyramids and the apical-bond anisotropy. XLD and the resulting analytical capabilities have been utilised in a microscopy context before, i.e., X-ray linear dichroism microscopy^16,29,30,37,60,61^, to provide a 2D spatially-resolved analysis tool. XLD microscopy and tomography at the current resolution level are particularly suited for the characterisation of materials with a net-orientation or anisotropy. Selected cubic materials, a large fraction of non-cubic crystalline materials, magnetic, ferroelectric and low-dimensional (e.g. graphene) materials, as well as selected coordination complexes and molecular arrangements / networks possess such local anisotropies in one form or another, and therefore display the required linear dichroism to some degree^16,39,41,43^.

**Supplementary Note 2: Electron density estimation of oxygen-vacancy rich V_2_O_5_:** The electron density, $\rho$, was calculated using tabulated density and molar density values according to the equation

| (S2) |
| --- |

$$\rho=\frac{\mathrm{Density}}{Molar Density}N_{A}* (Sum of Atomic Numbers in the Formula Unit)$$

Additionally, for oxygen-vacancy rich V_2_O_5_, the following equation was used

| (S3) |
| --- |

$$\rho=\frac{3.357g/cm^{3}}{181.862 g /\mathrm{mol}}6.022\times{10}^{23} (23*2+(5-x)*8)$$

where $N_{A}$ is the Avogadro constant and $x$ is the average number of oxygen vacancies per unit cell. From PXCT measurements, we determine the average number of oxygen vacancies per unit cell to be to 1.2. Given the additional annealing step, which triggers oxygen vacancy formation, the observed 1.2 oxygen vacancies per unit cell agrees with experimental reports of 1.14 oxygen vacancies per unit cell^92^. For the pre-experimental estimate of the electron density of oxygen-vacancy rich V_2_O_5_ (Table S1), 0.85 oxygen vacancies per unit cell were assumed based on a reference sample.

**Supplementary Note 3: XL-DOT composite material considerations and unit cell orientation:** For the examination of composite materials, consisting of multiple linear dichroism exhibiting materials and/or polymorphs, as for example encountered in biominerals or geological samples^37,93^, it might not be possible to retrieve the orientation field of each material from a single XL-DOT measurement. In this case, multiple XL-DOT measurements might be needed to retrieve the orientation field of each material to highest accuracy. While the electron density tomograms can be used for material identification and segmentation, XL-DOT measurements at different X-ray energies are needed to retrieve the orientation field of each material if their associated spectral features, which exhibit linear dichroism, are distinctly separated. Should the features fall within a couple eV of each other, and depending on the instrument’s energy resolution, a single XL-DOT measurement is sufficient to recover the orientation field of the different materials simultaneously^94^.

Since we performed XL-DOT at a single X-ray energy, only the *c*-axis orientation of V_2_O_5_ was probed, and the relative orientation of the crystallographic *ab* axes of the unit cells is undetermined. While this is sufficient to address several aspects of microstructural characterisation, the orientation determination of the secondary axes would be very helpful, for example, for the in-depth characterisation of crystallography defects such as dislocations^95^. For materials where the axes a ≠ b ≠ c, the full orientation tensor, could, for example, be obtained through the implementation of a second detector that simultaneously collects a low-resolution diffraction tomogram in order to refine the high-resolution XL-DOT dataset. For selected materials, a second option involves the collection of a spectroscopic XL-DOT data across an absorption edge. Such a spectroscopic measurement could benefit from sparsity as performed for X-ray transmission near-edge spectroscopic tomography^11,96^. The resulting spectroscopic XL-DOT datasets, which probe secondary anisotropies, would provide the desired orientation of the *ab* axes in tandem with a chemical characterisation of the defects.

**Supplementary Figure S1: Schematic illustrating the preparation of the sintered polycrystalline vanadium pentoxide sample.** To increase the V_2_O_5_ grain size and to assemble a stable porous structure from the initially loose powder, a mixture of V_2_O_5_ and polystyrene spheres was pressed into pellet shape with a 1.2 t axial load. The resulting pellet was then heated to 590ºC for 5 hours, to increase the grain size, sinter the structure and decompose the polystyrene as much as possible, allowing the structure to condense further.

**
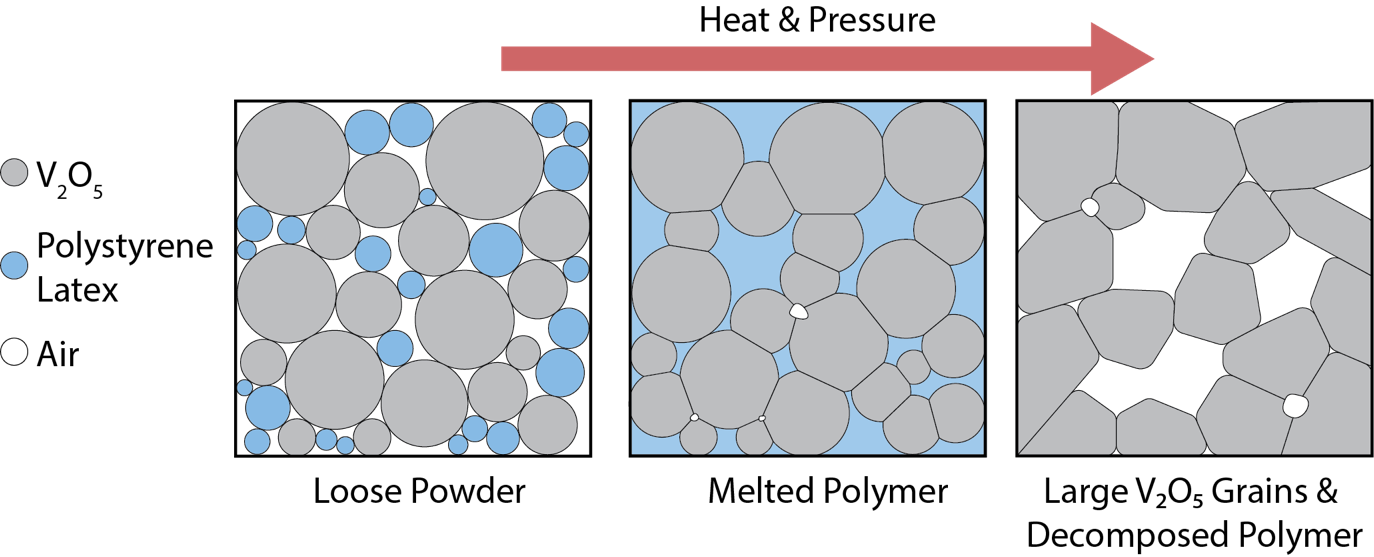
**

**Supplementary Figure S2: Powder X-ray diffraction of vanadium pentoxide.** Shown in black is PXRD data collected from V_2_O_5_ powder. Shown in orange is data collected from the sintered V_2_O_5_ pellet, prior to being manufactured into the sample pillar. All reflections can be assigned to orthorhombic V_2_O_5_^54^. Rietveld refinement^97^ using an anisotropic size-strain model^98^ suggests the average crystal in the sintered pellet to be asymmetric in shape, with a short axis coherence length of > 300 nm . Instrument broadening was taken into account. The shift observed after sintering is likely to be due to lattice contraction.

**
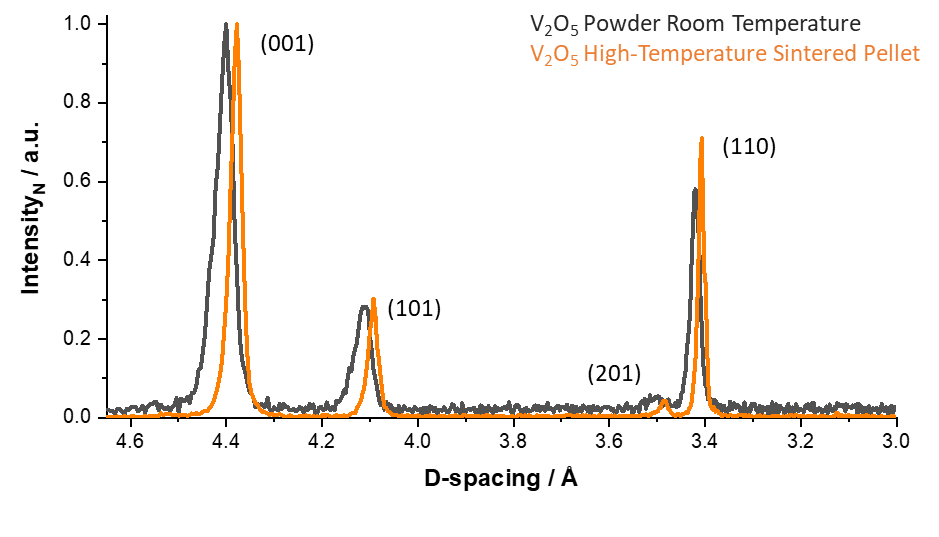
**

**Supplementary Figure S3: Images of the examined V_2_O_5_ pillar.** Shown is the progression of sample preparation including (a) a photograph of the fractured sintered pellet, and subsequent Scanning Electron Microscopy (SEM) images of (b) the micro-lathe pre-shaped pillar, (c) the FIB-milling reduced pillar, (d) the tomography-pin transfer and (e) the final mounted sample pillar. Scale bars are 1000 µm (a) and 20 µm (b-e).


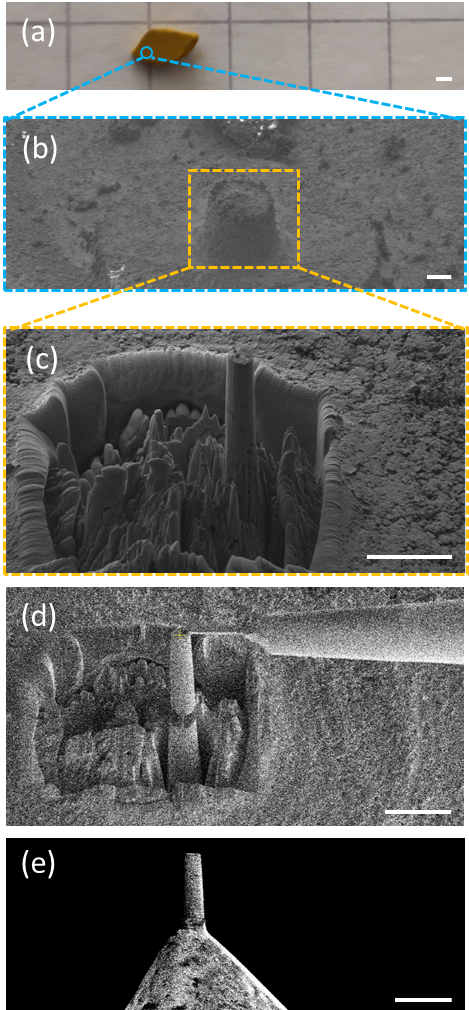


**Supplementary Figure S4: X-ray transmission near-edge spectra of V_2_O_5_.** Spectroscopic data was acquired using both LH and LV X-ray polarisations, showing both the energy and polarisation dependency of the X-ray linear dichroism of V_2_O_5_. Grey lines indicate the energies at which tomographic projection measurements were made for the off-resonance and on-resonance tomograms. On resonance, the linear dichroism effect can be observed in both amplitude and phase, as the sample-illumination interaction strength changes with polarisation. The optimal LD constrast in the phase and amplitude occur at different energies. The optimal energy for maximizing phase contrast was chosen to be 5.469 keV due to the higher resolution of individual projections. The full width at half maximum of the peak is approximately 3 eV, indicating that a higher energy resolution is not required to perform XL-DOT on this material.The projections measured at this energy were used for the reconstruction of the orientation tomogram. The tomographic projections measured at the indicated off-resonance energy (5.4 keV) were used for the reconstruction of the electron density. As this energy was sufficiently far from the pre-edge peak, which exhibits LD, we could safely assume that only the electron density contributed to the scattering factor.

**
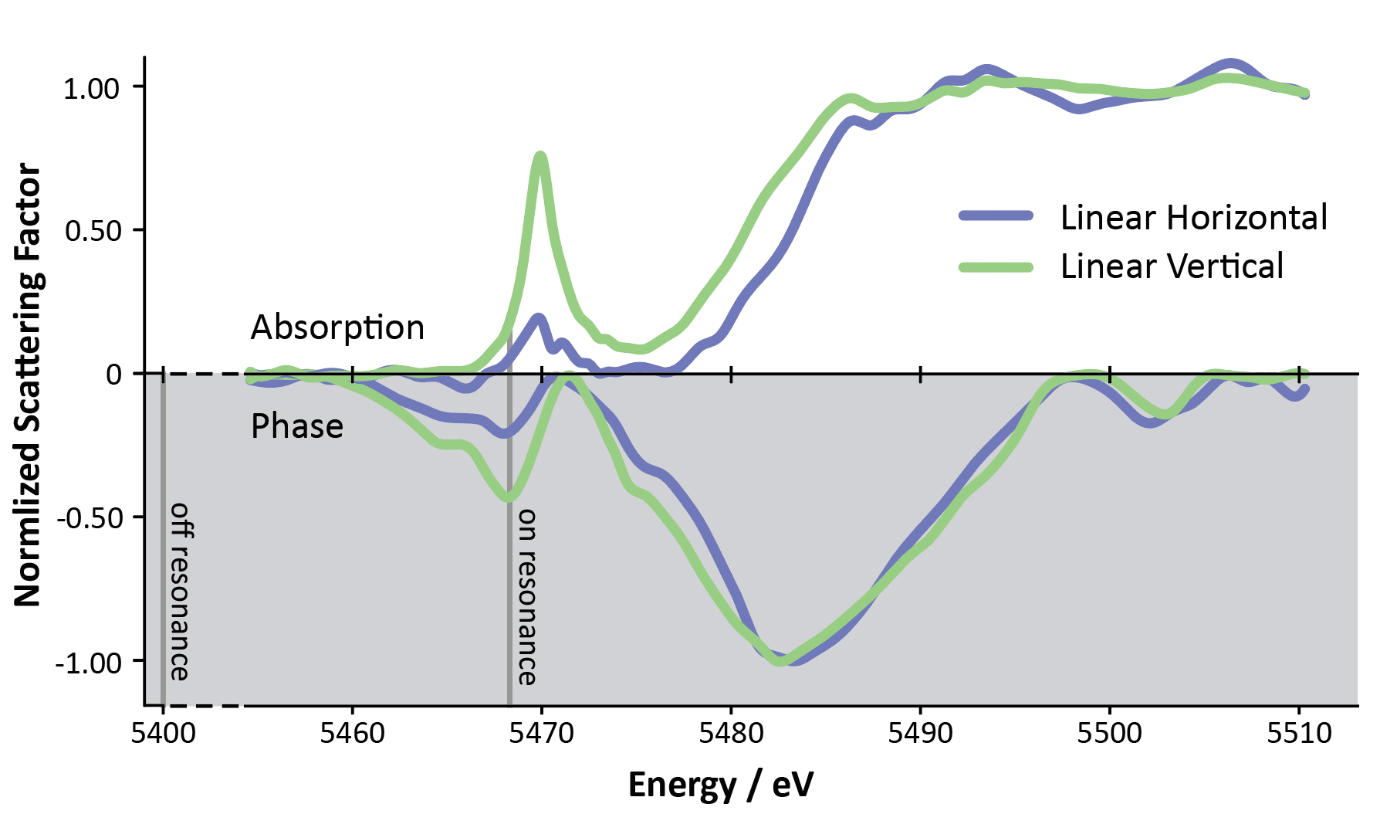
**

**Supplementary Figure S5: Comparison of ptychographic image reconstructions.** Typical reconstructed X‑ray ptychography images (tomographic projections). Comparison of amplitude (top, a-e) and phase (bottom, f‑j) reconstructions. (a, g) Reconstructions acquired at 5.400 keV, i.e., below the absorption edge and in the absence of vanadium linear dichroic contrast. Projections acquired at the vanadium pre-peak (5.469 keV) using a linear vertical (left) and linear horizontal (right) polarised illumination are shown in (b‑c, g‑h). The isolated LD signal is shown as the difference between LH and LV projections (d,i) and the difference between on resonance and off resonance projections for the same polarisation (e,j).


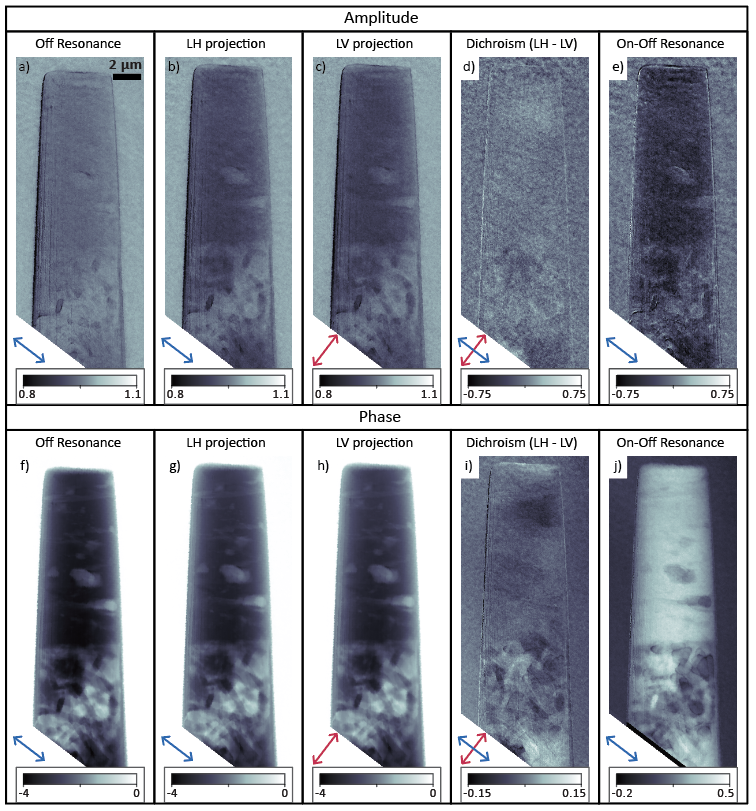


**Supplementary Figure S6: Spatial resolution of ptychographic image reconstructions.** Fourier ring correlation (FRCs) curves of phase image reconstructions. Correlation curves for (a) projections acquired at the absorption edge (at 5.469 keV) using linear horizontal polarisation and (b) below the absorption edge (at 5.400 keV). The former projections are the sum of electron density and linear dichroic contrast, while the latter projections comprise only electron density contrast. The half-pitch resolution is given by the intersection of the correlation curve with the 1‑bit threshold. In this case, no intersection implies single pixel resolution. The pixel size is 30.91 nm in (a) and 31.29 nm in (b).


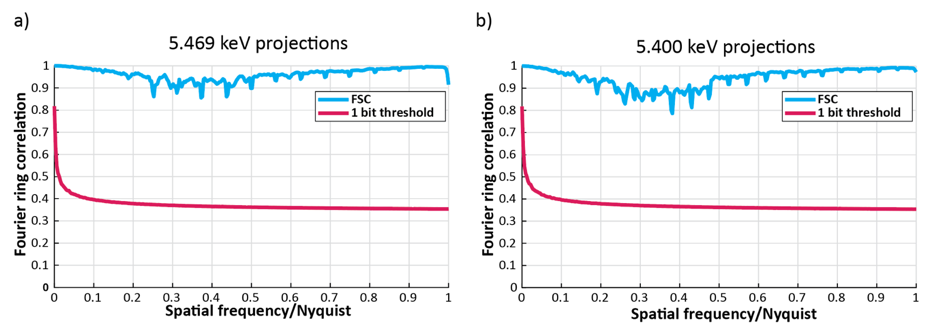


**Supplementary Figure S7: Schematic of the XL-DOT reconstruction process.** During the experiment, the sample (i) is used to acquire projections (ii) containing linear dichroic contrast at multiple angles and/or linear polarisation states. The collection of these projections (iii) constitutes the dataset necessary for the subsequent reconstruction process. The reconstruction algorithm begins with an initial guess of the internal structure of the sample (a). For each experimentally obtained projection, a corresponding projection is simulated (b), using the same measurement parameters as in the experiment. The stack of simulated projections (c) and the experimentally obtained projections (iii) are then used to evaluate the error metric (d) according to Equation (4). This metric quantifies how well the guess structure can replicate the experimentally obtained dataset. The objective is to correct the guess structure, (a), such that the error metric is reduced, indicating a closer match between the guess and the internal structure of the measured sample. This is achieved through the implementation of gradient descent. Having calculated the analytical gradient (e) according to Equation (5), a corresponding correction (f) – conventionally a scalar multiple of the gradient – is applied to the test object. This ensures a reduction in the error metric. The iterative repetition of this process continues for a set number of iterations or until a convergence criterion is met, ultimately yielding the XL-DOT result.

~~
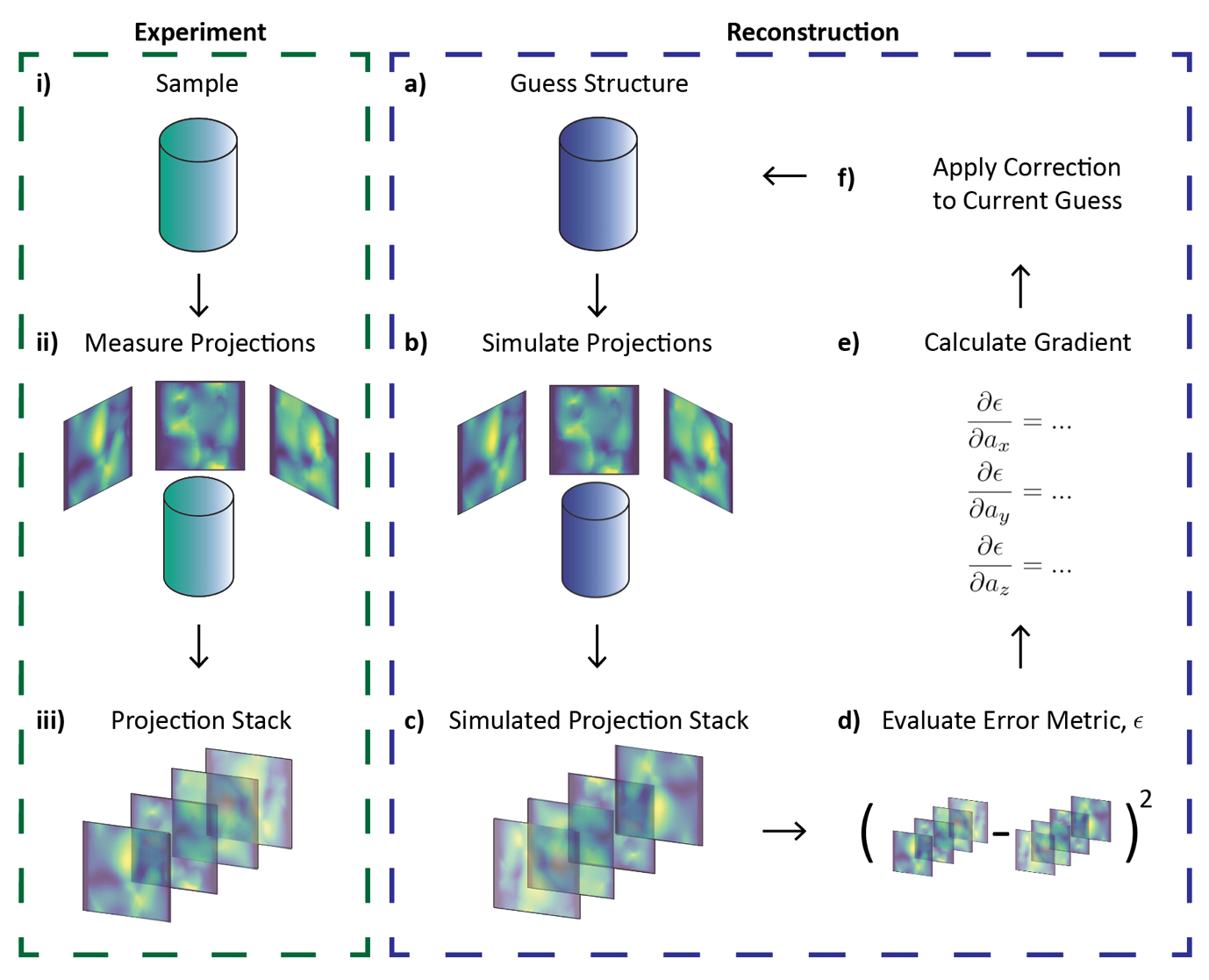
~~

**Supplementary Figure S8: Spatial resolution of the XL-DOT reconstruction.** a-d) Estimating resolution from the ability to resolve sharp features. a) Orientation view of chosen sharp boundary (running from top to bottom in the middle of the image) at which the resolution was evaluated. b) Isolation of the out-of-plane component and c) magnified image of the boundary. For each horizontal line of pixels across the boundary in c), a line profile was obtained from which the 10%-90% edge criteria were calculated, with the corresponding value shown on the plot to the right of the image. The red dashed line indicates the average resolution of 73 nm. d) Example of one such line profile, taken from the dashed grey line in c). The edge jump, ∆, is the largest difference in elevation across the boundary, and the resolution of the XL-DOT reconstruction is given by the difference between the 10% ∆ and 90% ∆ distances. The spatial resolution was also calculated for a smaller grain, shown in e) orientation view and f) equivalent x-y azimuthal angle plot. g,h) Line profiles corresponding to the lines of the same colour in (f) demonstrating 47 nm to 65 nm spatial resolution, similar to the resolution of the larger grains in (a-d). Also provided are Fourier shell correlation (FSC) curves (i-k) of each orientation scalar component (x, y, z), respectively. The half-pitch resolution is given by the intersection of the correlation curves with the half-bit threshold and provided in the figure. The voxel size is 30.91 nm.


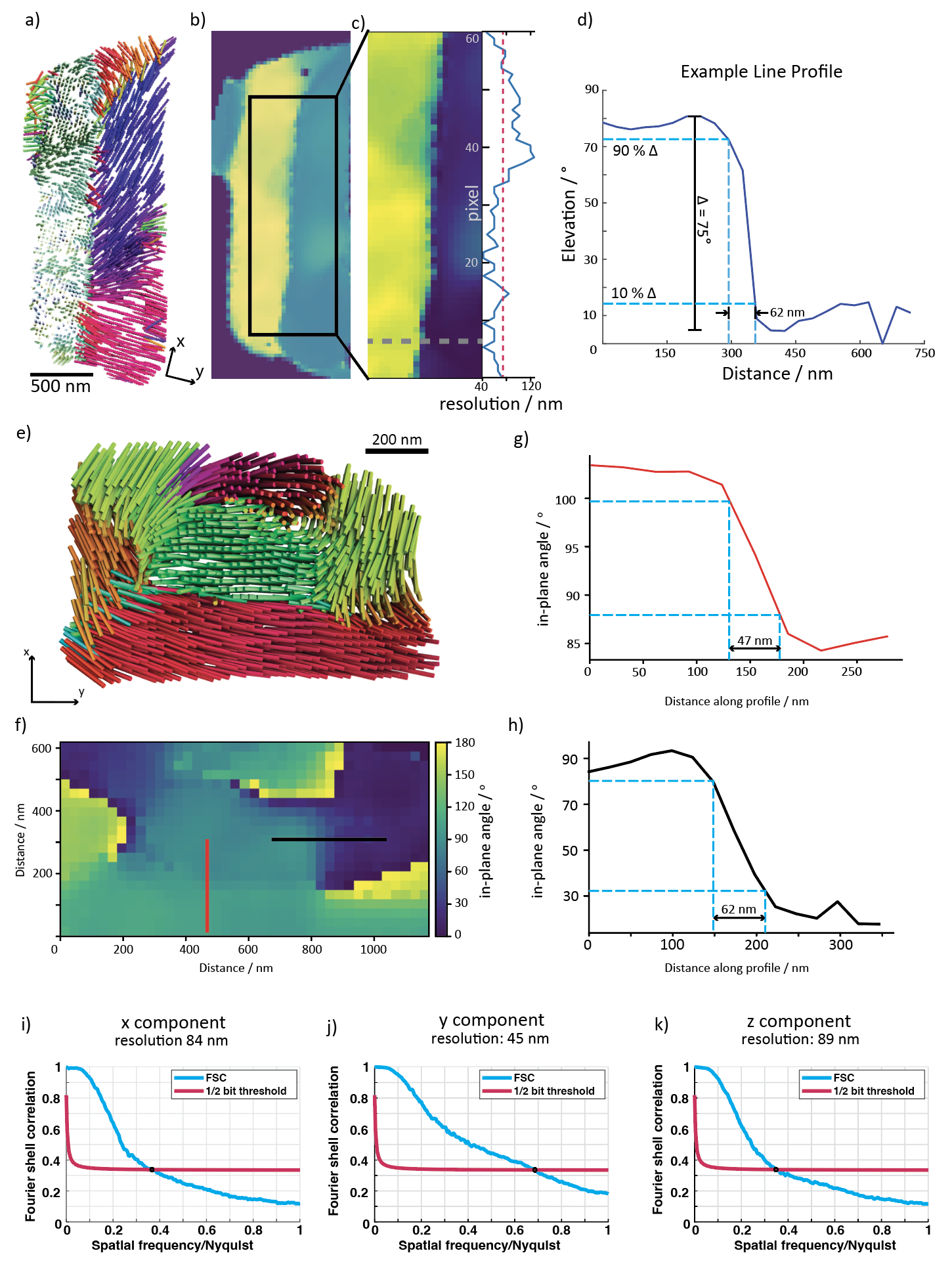


**Supplementary Figure S9: Electron density tomogram of the examined sample pillar.** (a) A volume rendering of the acquired electron density tomogram. The voxel volume is (30.91 nm)^3^. The red box indicates the volume considered for microstructural examination. (b) Electron density histogram of the volume shown in (a). The expected electron densities of the t, defect-rich V_2_O_5_ and pristine V_2_O_5_ are indicated with markers (i), (ii) and (iii), respectively. Depicted by the horizontal bar are the upper- and lower-bounds used for the isolation of V_2_O_5_. (c) Slice through the electron density tomogram, with regions of polymer (i) and the defect-rich V_2_O_5_ (ii) labelled accordingly. The increased electron density present on the outermost layers of the pillar (∼90 nm-thick) was attributed mainly to gallium implantation (but also amorphization and material redeposition), which resulted from the FIB milling during sample preparation. As gallium has a higher electron density than V_2_O_5_ (see Table S1), the electron density of the affected area is greater than in the central parts of the sample, which remained unaffected.

**
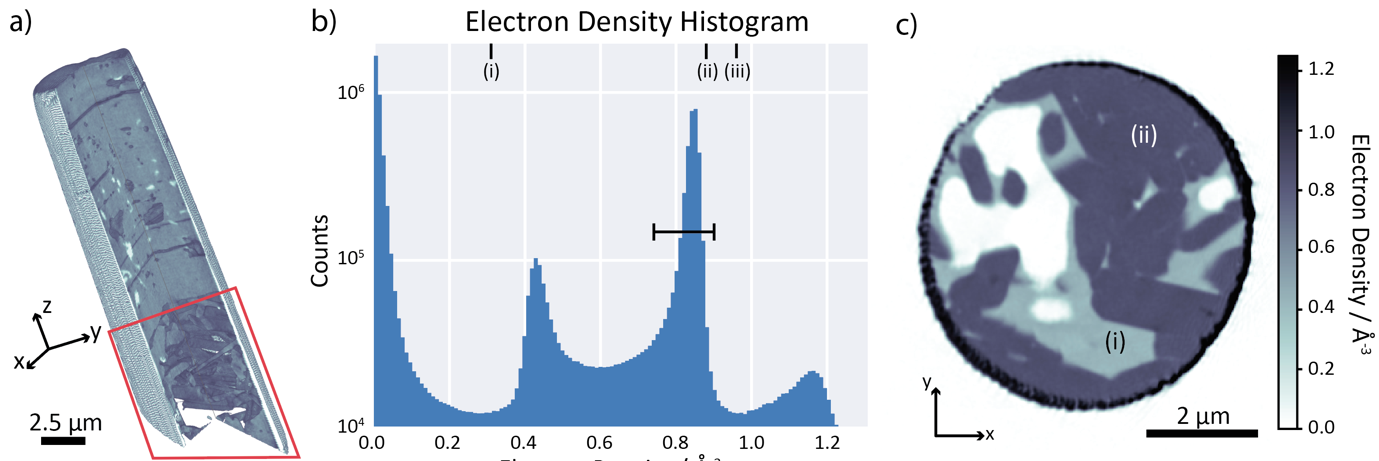
**

**Supplementary Figure S10: Spatial resolution of the electron density tomogram.** Fourier shell correlation (FSC) curve of the electron density tomogram derived from the phase projections acquired with the X-rays tuned to an energy below the absorption edge (5.400 keV). The spatial resolution is given by the intersection of the correlation curve with the half-bit threshold. The voxel size is 31.29 nm, and the spatial resolution is 44 nm.


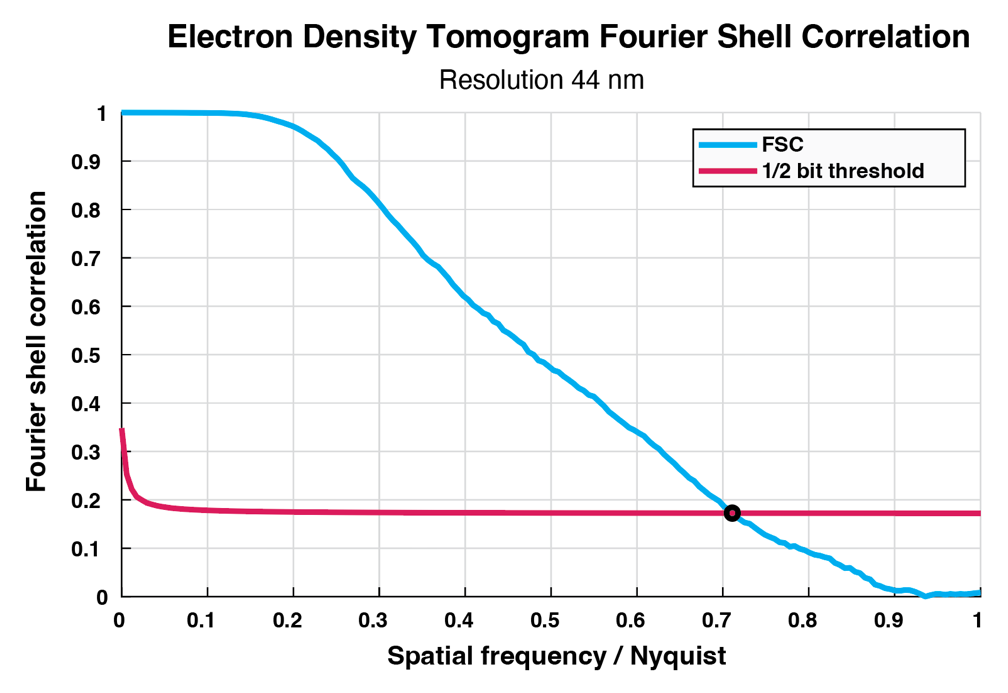


**Supplementary Figure S11: Error in the determination of the orientation.** Volume rendering of an isolated V_2_O_5_ grain, exhibiting minimal variations in (a) c-axis orientation and (b) electron density, so that we can assume that this is a single crystal grain. (c) Histogram showing the voxel-level measured orientation spread in this region. From this we determine the following parameters: mean electron density: 0.84 A^-3^, standard deviation of electron density: 0.024 A^-3^, Length: 3.2 μm, Azimuthal angle (in-plane, xy-angle): -70 ± 10˚, Elevation (out-of-plane angle): 24 ± 8˚.

**
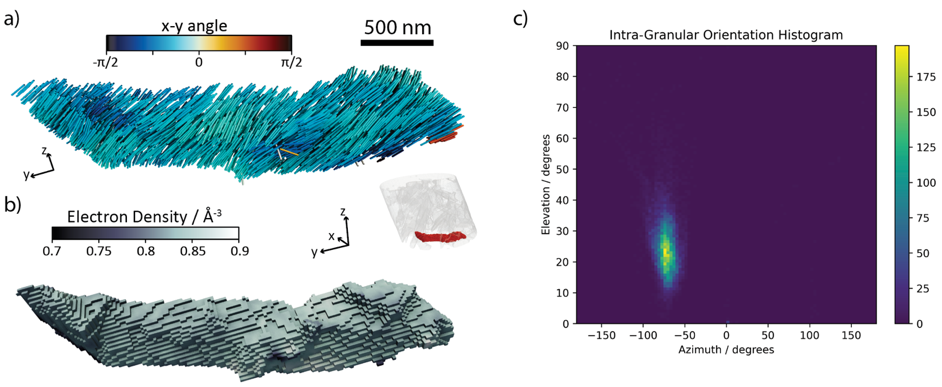
**

**Supplementary Figure S12: Validation and comparison of XL-DOT reconstructions.** (a) Comparison of reconstructed orientation tomograms obtained from the average of 5, 10, 40 reconstructions from different random-valued starting guesses. Provided are virtual cuts through the averaged tomogram. The voxel size is 30.91 nm and figures share a common greyscale as indicated. The same cut, but this time of the scalar reconstruction of the projections obtained using linear vertical polarisation, which is used for validation, is shown on the right for comparison. The contrast of the scalar reference originates from the out-of-plane component of the *c*-axis orientation according to Equation (6). (b) Graph of the angular uncertainty versus the number of averaged reconstructions. The inset is a magnification of the angular uncertainty between 15 and 40 averages, emphasizing the small improvement in angular uncertainty (~0.25°). As a compromise between computational and time constraints, and small improvements to the reconstruction, the final reconstruction was calculated from an average of 40 reconstructions.


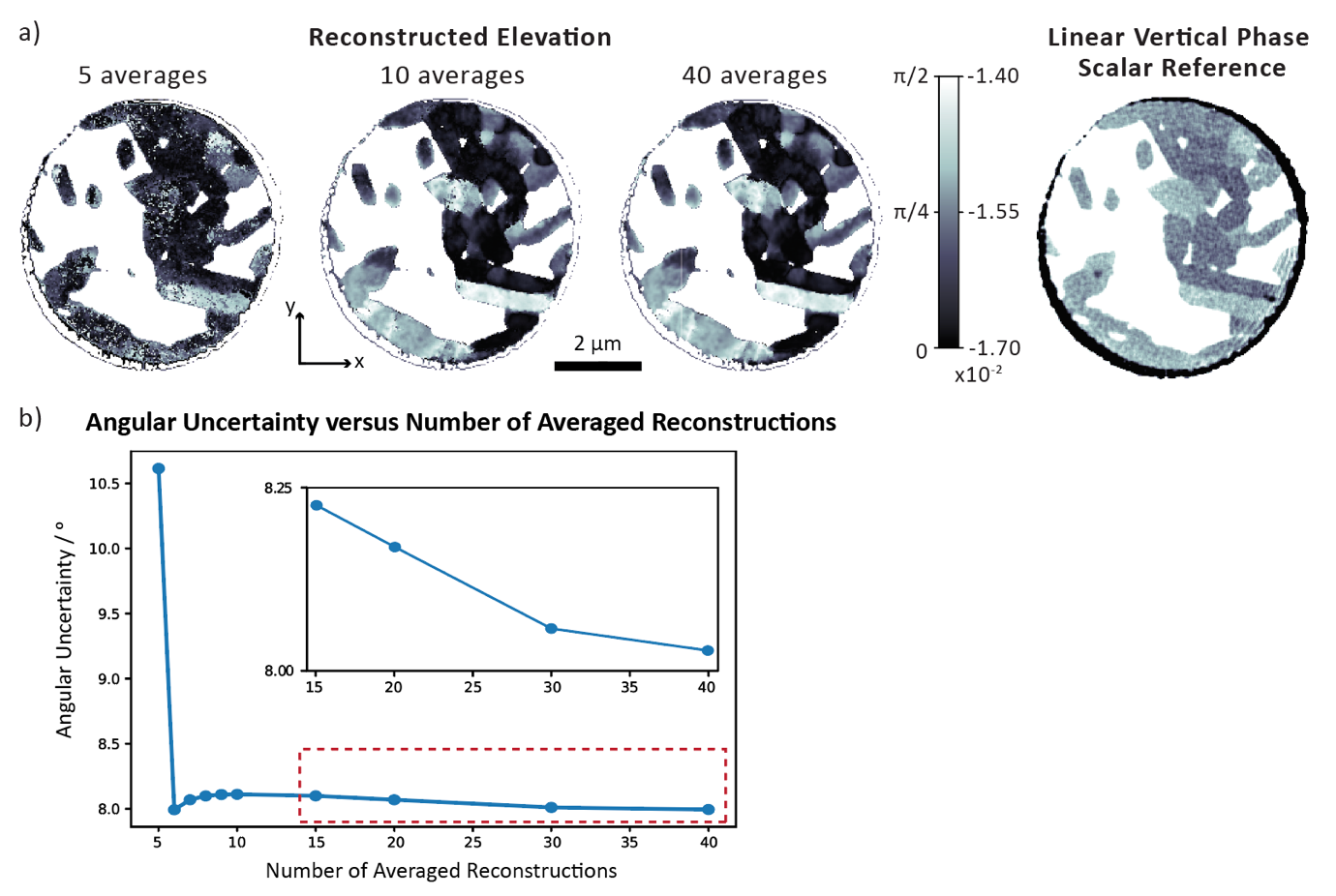


**Supplementary Figure S13: Inter-granular characterisation of V_2_O_5_ domains.** Histograms showing the correlation between grain volume, grain average electron density, mean length, and sphericity (where 0 and 1 represent an infinite rod and a perfect sphere, respectively). The occurrence frequency histograms are given on the diagonal, with the blue lines corresponding to the kernel density. The upper-right plots (within the orange boundary) are the bi-variate histograms corresponding to the two properties labelled on the vertical and horizontal axes. In the lower-left (within the red boundary), the kernel density estimate (KDE) plots are shown, with the Spearman correlation coefficients between the corresponding grain properties displayed.


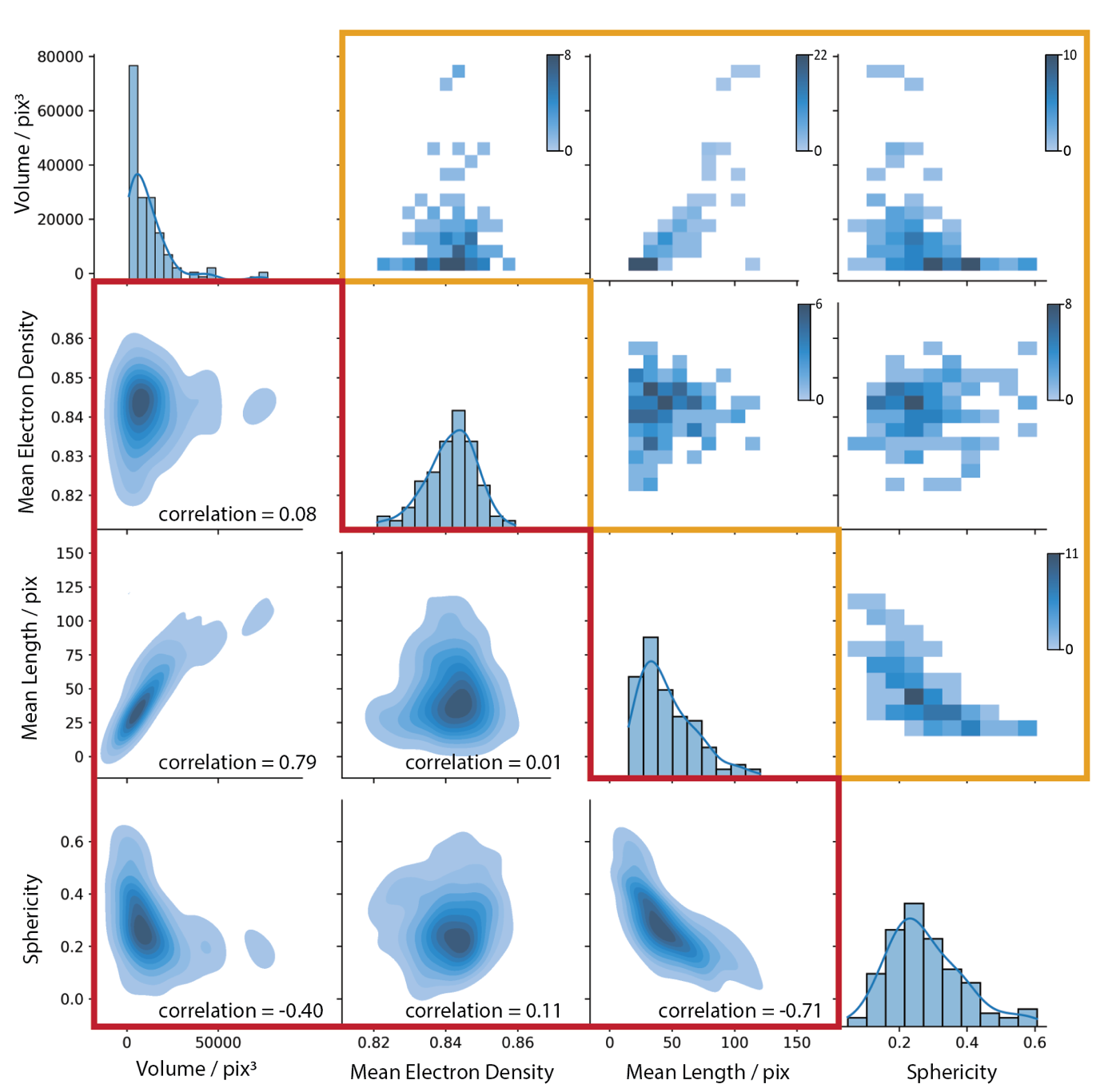


**Supplementary Figure S14: Impact of the number of tilts on the reconstruction quality.** a) Plot of angular error obtained from numerical simulations of the XL-DOT technique against number of tilt axes. The shaded areas represent the 5^th^ to 95^th^ percentile errors (light grey) and 25^th^ to 75^th^ percentile errors (dark grey). The red data points (line is a guide to the eye) represent the mean angular error. From the plot it is evident that four tilts or more yield optimal results, while a preliminary reconstruction can be obtained from as few as two tilts. Slices through the XL-DOT reconstructed sample volume obtained from b) two-tilt and c) four-tilt reconstructions. Although the reconstruction with only two tilt axes has an increased angular uncertainty, lower spatial resolution, and is prone to artefacts, there appears to be an overall agreement in terms of the reconstructed orientation with the four-tilt reconstruction.

**
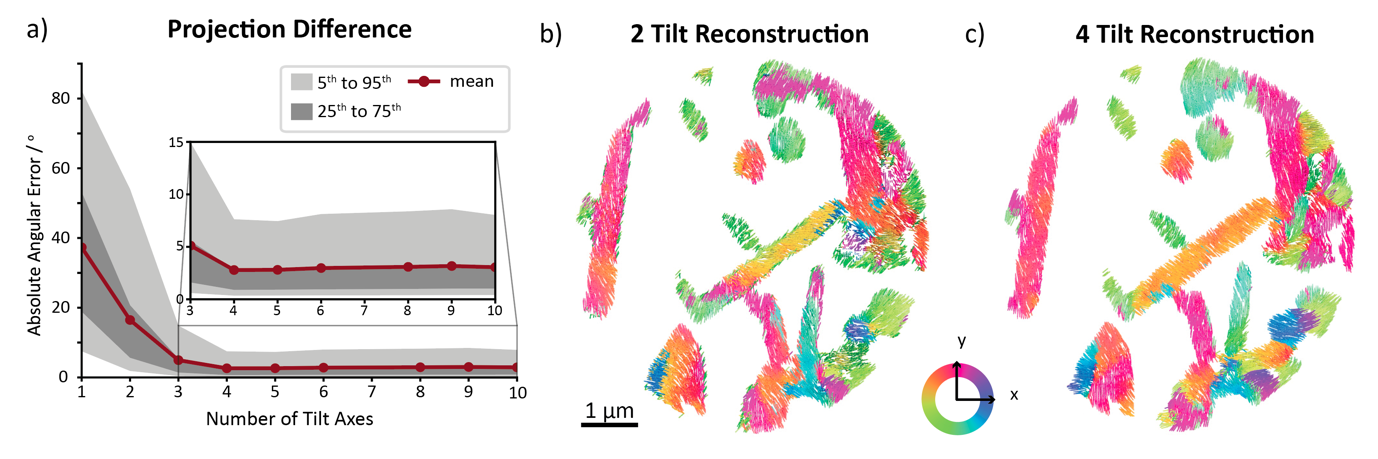
**

**Supplementary Table S1: Electron densities of reference components.** Electron densities of relevant materials, including other oxides of Vanadium that V_2_O_5_ could decompose to. Polystyrene polymer was used in the sintering, leading to the porous morphology of the pillar after decomposition. Gallium is also included so that Ga implantation from the FIB processing can be identified and excluded from the analysis. Electron densities were calculated using tabulated molecular weight and mass density values^32^. * See Supplementary Note 2.

| Compound | Electron Density / Å^-3^ |
| --- | --- |
| V_2_O_3_ | **1.37** |
| VO_2_ | **1.29** |
| V_2_O_5_ | **0.96** |
| V_2_O_5_ (Oxygen Vacancy-Rich) * | **~0.88** |
|  |  |
| Air / Pores | **0.003** |
| Polystyrene (~C_8_H_8_) | **0.31** |
| Ga | **1.6** |
